# Comprehensive transcriptome of muscle development in Sichuan white rabbit

**DOI:** 10.1186/s12863-025-01322-5

**Published:** 2025-04-23

**Authors:** Xiangyu Zhang, Kai Zhang, Dengping Huang, Shangjun Yang, Min Zhang, Qin Yin

**Affiliations:** 1 Sichuan Academy of Science Academy, Chengdu, 610066 China; 2 Animal Breeding and Genetics Key Laboratory of Sichuan Province, Chengdu, 610066 China; 3 Sichuan Academy of Grassland Sciences, Chengdu, 611743 China

**Keywords:** Sichuan white rabbit, Muscle development, mRNA, Small RNA, lncRNA

## Abstract

**Background:**

The Sichuan white rabbit is a unique domestic breed and is famous for its high meat production. Muscle development is a complicated biological process, but the underlying regulatory mechanisms have not been elucidated. Here, we generated comprehensive transcriptome datasets (i.e., mRNAs, miRNAs and lncRNAs) in three developmental stages of Sichuan white rabbits, and aim to systematically explore the regulatory network in myogenesis.

**Results:**

We generated extensive transcriptome datasets (mRNAs, miRNAs and lncRNAs) revealing the myogenic regulatory network at different time points. Our differential expression analysis identified 2,995 DE genes, 1,211 DE-lncRNAs, and 305 DE-miRNAs with distinct expression patterns across developmental stages. In addition, functional enrichment analysis of DE mRNAs and miRNAs indicates their involvement in muscle growth, development, and regeneration, highlighting biological processes and muscle-specific functions. Interaction analysis between DE-lncRNAs and mRNAs uncovered a complex regulatory network, especially between 21 and 27 days of development. These findings contribute to better understanding of the transcriptomic changes during muscle development and have implications for breeding improvement in Sichuan white rabbits.

**Conclusions:**

Our study provides a comprehensive overview of the transcriptomic changes during muscle development in Sichuan white rabbits. The identification and functional annotation of DE genes, miRNAs, and lncRNAs provide valuable insights into the molecular mechanisms underlying this process. These findings pave the way for targeted investigations into the role of non-coding RNAs in muscle biology.

**Supplementary Information:**

The online version contains supplementary material available at 10.1186/s12863-025-01322-5.

## Background

Rabbit meat becomes increasingly popular in China because of its remarkable nutritional value and unique flavour. On the one hand, rabbit meat has moderately high energy values, low fat contents, and low cholesterol levels [1] and greatly satisfies modern consumers’ desire for a healthy lifestyle. On the other hand, the delectable texture and taste of rabbit meat make it more prevalent worldwide, especially in China. As the largest rabbit meat producer worldwide, Chinese rabbit meat production has steadily increased from 690,000 tons in 2010 to 849,150 tons in 2016 (http://www.fao.org/faostat/en/#data/QL). China has various domesticated rabbit breeds, while most local breeds achieve a low meat production rate [2]. Sichuan white rabbit, due to its high-yield meat production, strong adaptability and fecundity [2, 3], has become one of the well-known breeds in China. Thus, improving the quality and production of Sichuan white rabbits is critical for breeding improvement.

Muscle is the largest organ of body mass in humans and other animals, and its functions include movement, postural support and thermogenesis [4]. The development of muscle is a complicated biological process that includes distinct embryonic and postnatal phases. Many diffusible signaling molecules, transcription factors and non-coding RNAs [e.g., microRNAs (miRNAs) and long non-coding RNAs (lncRNAs)] that contribute to muscle development have been identified. These regulators serve as direct templates for protein synthesis, which is fundamental to the growth and repair of muscle fibers [5]. In vertebrate embryos, numerous miRNAs are expressed in the developing somites of zebrafish [6], Xenopus [7] and chicks [8]. Muscle-specific mRNAs, such as miR- 1 and miR- 133, which are known to regulate muscle differentiation by targeting specific transcription factors and structural proteins, can regulate muscle maturation [9]. In addition, lncRNAs can act as scaffolds for certain regulatory proteins or be involved in the regulation of other non-coding RNAs [10–12]. Previous studies reported that lncRNAs associated with myogenesis include steroid receptor RNA activator (Sra), which co-activates *MyoD* [13], and LncMyoD, which is itself activated by *MyoD*, together with linc-MD1, which regulates miR- 133 to further enhance differentiation [14]. In addition, a novel lncRNA lncMGR, which promotes myoblast differentiation and muscle fiber hypertrophy, can recruit cyclin-dependent kinase 9 (*CDK9*) and sponge miRNAs, such as miR- 2131 - 5p, to regulate the expression of skeletal muscle myosin heavy chain 1 A (*MYH1 A*) [4].

Current studies are limited to the regulation of either mRNAs or miRNAs related to the proliferation and differentiation of skeletal muscle cells. Thus, the identification of more potential muscle-associated protein-coding genes (i.e., mRNAs) and non-coding RNAs (e.g., miRNAs and lncRNAs) could better dissect the underlying regulatory mechanisms. In this study, we generated comprehensive transcriptome datasets (i.e., mRNAs, miRNAs and lncRNAs) in different developmental stages of Sichuan white rabbit and aimed to systematically explore the regulatory network in myogenesis. Our study reveals an epigenetic-mediated myogenic regulatory mechanism and provides insights into the roles of non-coding RNA in myogenesis.

## Methods

### Animals and sample collection

**Fig. 1:**
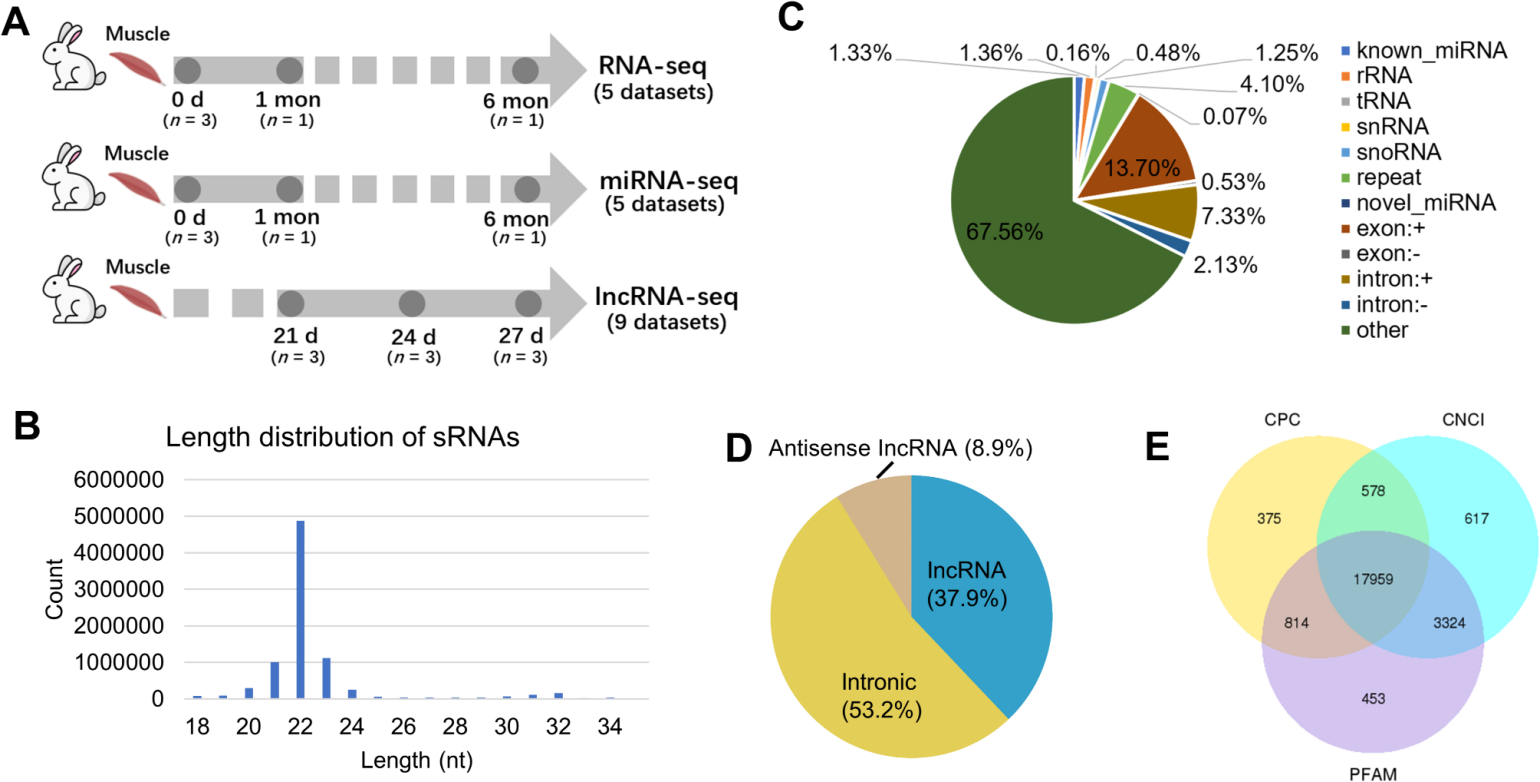
Experimental design and overview of the data. **A** Schematic diagram of the experiment and data generation. **B** Length distribution of sRNAs. **C** Classification of miRNAs. Colors represent different types of sRNAs. **D** Classification of lncRNAs. Colors represent different types of genomic annotations. **E** The intersection of lncRNAs predicted by the coding potential calculator (CPC), coding-non-coding index (CNCI) and protein families database (PFAM)

The Sichuan white rabbit (SWR) used in this study were raised on the farm of Sichuan Animal Sciences Academy (Chengdu, Sichuan Province) under standard and uniform housing conditions (temperature: 22–26 °C; humidity: 60–70%). All the animals were healthy male rabbits (i.e., siblings) with similar body conditions. The animals were fed twice per day with formula diets containing 1.2% crude protein, 16% crude fiber, 8% crushed ash, 0.6% calcium, 1.2% lysine, 0.4% phosphorus, and 0.6% sodium chloride and had *ad libitum* access to water. The longissimus dorsi of rabbits is no longer expressed 27 days after delivery. For long non-coding RNA sequencing, longissimus muscle tissues were collected at 21 days, 24 days and 27 days after birth, with three biological replicates for each time point. Hereinafter, we used “21 d”, “24 d” and “27 d” to represent the above time points (Fig. 1A). For the mRNA and miRNA sequencing samples, muscle tissues were collected at 0 day, 1 month and 6 months after delivery to represent the fetal, child and adult stages separately, and were marked as “0 d”, “1 mon” and “6 mon” hereinafter. Due to the limitations of sample collection, we obtained three biological replicates for 0 d, one biological replicate for 1 month and one biological replicate for 6 months (Fig. 1A). Muscle tissues were collected immediately after slaughter. The tissues were cut into small pieces and rinsed with PBS. The collected tissues were stored in liquid nitrogen and then transferred to − 80 °C for subsequent high-throughput sequencing.

### RNA extraction, library construction and sequencing

Total RNA from muscle was extracted using TRIzol reagent (Thermo Fisher Scientific, Waltham, MA, USA) according to the manufacturer’s protocol. After purification, the quality was checked via agarose gel electrophoresis and a NanoPhotometer^®^ spectrophotometer (IMPLEN, CA, USA). The RNA was measured with an Agilent 2100 RNA 6000 Nano Kit (Agilent Technologies, Waldbronn, Germany). All the samples had high-quality RNA with an RNA integrity number (RIN) > 6. For mRNAs and lncRNAs, strand-specific sequencing libraries were constructed via the ribosomal RNA (rRNA) removal method following the manufacturer’s instructions using the Illumina Standard RNA Sample Library Preparation Kit (Illumina, San Diego, CA, USA). For miRNAs, RNA molecules ranging from 18–30 nt in size were enriched from total RNA by the polyacrylamide gel electrophoresis (PAGE). The 3′ adaptors were then added, followed by enrichment of RNAs with lengths of 36–44 nt and ligation of 5’ adaptors to the RNAs. RNAs were converted to cDNA, and PCR amplification was performed for library construction. All the above libraries were sequenced on the Illumina NovaSeq 6000 platform, and generated 150-bp paired-end (PE150) reads.

### Data pre-processing

All the raw RNA-seq (i.e., mRNA, miRNA and lncRNA) reads were filtered using Trimmomatic (v0.36) software [15]. Specifically, adapters and reads of low quality, in which more than half of the bases had quality < = 20 or more than 10% of the bases were missing (Ns > 10%) were discarded to obtain clean reads. The clean reads were assessed with FastQC (v0.11.9) (https://www.bioinformatics.babraham.ac.uk/projects/fastqc/) for quality check.

For the mRNA sequencing reads, the clean reads were mapped to the rabbit reference genome *OryCun2.0* (GCA_000003625.1) by HISAT2 software (v2.1.0) [16] with the following parameters: −n-ceil: L, 0, 0.15; −mp: MX = 6, MN = 2; −np: 1; −rdg: 5, 3; −efg: 5, 3; −score-min: L, 0, − 0.2. Mapped reads were quantified with the FeatureCounts program from the Subread suite (v2.0) [17]. The raw counts of genes were normalized to transcripts per million (TPM) with the in-house script for subsequent analysis.

For miRNA sequencing, clean reads were aligned to the rabbit (*OryCun2.0*) mature miRNA database in miRBase (v20) [18] and matched with known miRNAs, ribosomal RNA (rRNA), small nuclear RNA (snRNA), small nucleolar RNA (snoRNA), and transfer RNA (tRNA) sequences. miRNA abundance was quantified as counts and normalized to the TPM. Unmapped clean reads were further aligned to the rabbit reference genome *OryCun2.0* using bowtie2 (v2.4.4) [19] software. To remove tags derived from protein-coding genes, repeat sequences, ncRNA, rRNA, tRNA, snRNA, and snoRNA, small RNA tags were mapped to RepeatMasker software [20] and the Rfam database. The distribution of the alignments was summarized using the software miREvo [21]. Thek novel miRNAs were predicted by miRDeep2 [22].

For the lncRNA sequencing reads, the clean reads were mapped to the rabbit reference genome via the HISAT2 (v2.1.0) program [16]. Then alignments were transferred to StringTie (v1.3.3) [23] and Cuffcompare (v2.2.1) [24] software for transcript assembly.

### Differential expression analysis

For each type of RNA-seq data in this study, we performed differential expression analysis via the R package DESeq2 (v1.32.0) [25] to identify differentially expressed (DE) genes, miRNAs and lncRNAs between time points (mRNA-seq: 0 d vs. 1 mon, 0 d vs. 6 mon, and 1 mon vs. 6 mon; miRNA-seq: 0 d vs. 1 mon, 0 d vs. 6 mon, and 1 mon vs. 6 mon; lncRNA-seq: 21 d vs. 24 d, 21 d vs. 27 d, and 24 d vs. 27 d). In each comparison, the former group (e.g., “0 d” in “0 d vs. 1 mon”) was used as the control group when we mentioned up-regulated and down-regulated expressions/genes. The matrix of normalized TPM was used for DE analysis. The *P*-values were corrected via the Benjamini–Hochberg method. Corrected *P*-value < 0.05 and |log_2_FC| > 1 were set as the thresholds.

### Target gene prediction for differentially expressed MiRNAs and LncRNAs

Target gene prediction was performed between three groups of differentially expressed miRNAs and lncRNAs. The prediction of target genes of miRNAs was performed by miRanda [26]. For DE lncRNAs, target genes were predicted by the positional relationship (co-location) and expression correlation (co-expression) of lncRNAs with protein-coding genes. lncRNA–mRNA co-regulated pairs (Pearson’s correlation coefficient > 0.8 and *P*- value < 0.05) were screened for Gene Ontology (GO) analysis.

### Functional enrichment analysis

For DE RNAs (i.e., mRNAs, miRNAs and lncRNAs) from all comparisons, we conducted Gene Ontology (GO) enrichment analysis with the R package Goseq (v3.19) [27]. We built a reference database for rabbits with the *Oryctolagus_cuniculus* GTF file (Ensembl genome browser 113). Additionally, Kyoto Encyclopedia of Genes and Genomes (KEGG) pathway analysis was also performed by KOBAS (https://bio.tools/kobas). *P*-values were calculated by hyper-geometric test. The above biological processes and pathways were considered statistically significant with *P*-values < 0.05.

## Results

### Summary of the mRNA, MiRNA and LncRNA sequencing data

A total of 19 RNA sequencing datasets were generated in this study, including 5 mRNA-seq, 5 miRNA-seq and 9 lncRNA-seq datasets. Each sequencing data contained 3 developmental time points (Fig. 1A, Table S1). After the quality control of the raw reads, we obtained 282,575,600 mapped paired-end reads with an average mapping rate of 85.49% (ranging from 82.52 to 90.28%) for the mRNA datasets (Table S2), 38,485,181 mapped paired-end reads with an average mapping rate of 91.72% (ranging from 85.36 to 95.97%) for the miRNA datasets (Table S3) and 794,268,228 mapped paired-end reads with an average mapping rate of 90.51% (ranging from 89.37 to 91.21%) for the lncRNA datasets (Table S4). In addition, the length of all the miRNAs was 18–35 nt, most of which were 21–23 nt (Fig. 1B), and rRNA accounted for less than 1.35% (Fig. 1C). The classification of lncRNAs is shown in Fig. 1D, with 48.7% of the reads were lncRNAs (i.e., antisense lncRNAs and lncRNAs). We identified 17,959 lncRNA transcripts from the intersection of the coding potential calculator (CPC), coding-non-coding index (CNCI), and protein families database (Pfam) (Fig. 1E).

### Differential expression analysis of mRNAs, MiRNAs and LncRNAs

**Fig. 2:**
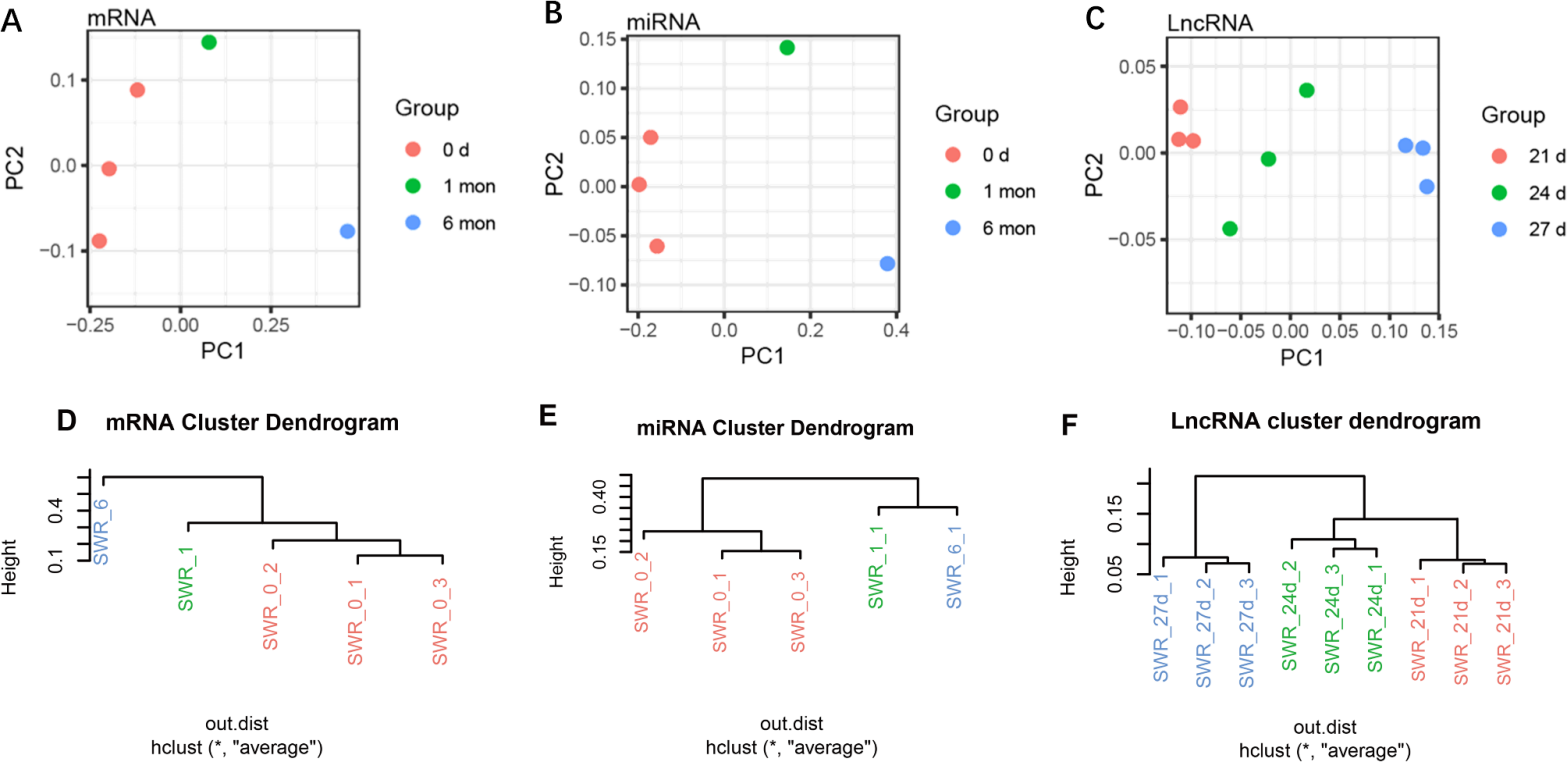
Principal component analysis (PCA) (**A**-**C**) and hierarchical cluster analysis (HCA) (**D**-**F**) of samples in mRNA, miRNA and lncRNA sequencing

To explore the characteristics of different developmental stages, we first verified the reliability of the identified RNAs with principal component analysis (PCA) and hierarchical cluster analysis (HCA) (Fig. 2). Although the sample size is limited, samples in the same group cluster together roughly. Next, we performed differential expression analysis between groups for each sequencing dataset separately. The former group (e.g., “0 d” in “0 d vs. 1 mon”) was used as the control group when we mentioned up-regulated and down-regulated expressions/genes.

**Fig. 3:**
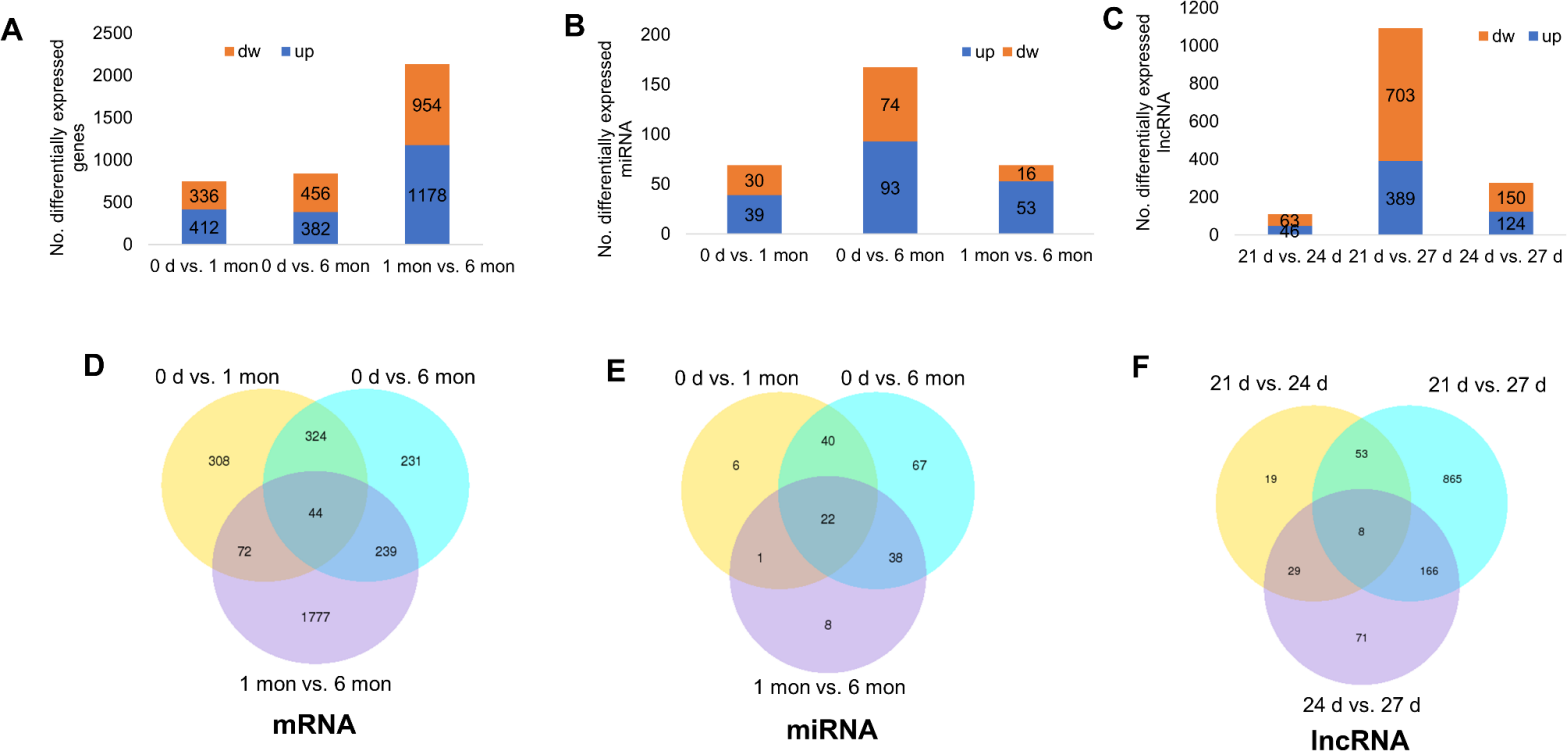
Differentially expressed DE genes, DE-miRNAs and DE-lncRNAs between different developmental stages. **A**-**C** The number of up- and down-regulated DEGs (**A**), DE-miRNAs (**B**) and DE-lncRNAs (**C**) in each pairwise comparison. **D**-**F** Venn diagram of DEGs (**D**), DEMs (**E**) and DELs (**F**) in each pairwise comparison

Among the comparisons, we detected a total of 2,995 differentially expressed (DE) genes from RNA-seq, 1,211 DE-lncRNAs from lncRNA-seq and 305 DE-miRNAs from miRNA-seq (Fig. 3A-C). For mRNA-seq, 44 genes were detected in all three comparisons, whereas 308, 231 and 1,777 DEGs were exclusively differentially expressed in the comparison of “0 d vs. 1 mon”, “1 mon vs. 6 mon” and “0 d vs. 1 mon”, respectively (Fig. 3D). Besides, there was small difference in the number of up and down-regulated DEGs across the three comparisons (Fig. 3A). For miRNA-seq, we detected the greatest number of DEGs in the comparison of “0 d vs. 6 mon”, followed by “0 d vs. 1 mon” and “1 mon vs. 6 mon” (Fig. 3B), which included 93 and 74 DE-miRNAs that were up- and down-regulated in the comparison of “0 d vs. 6 mon”, 39 and 30 DE-miRNAs that were up- and down-regulated in the comparison of “0 d vs. 1 mon”, and 53 and 16 DE-miRNAs that were up- and down-regulated in the comparison of “1 mon vs. 6 mon” (Fig. 3B). The greatest number of unique DE-miRNAs was detected in the comparison of “0 d vs. 6 mon”, followed by “1 mon vs. 6 mon” and “0 d vs. 1 mon” (Fig. 3E). We also observed that the number of up-regulated DE-miRNAs was generally greater than that of down-regulated DE-miRNAs (Fig. 3B). In terms of lncRNA-seq, we found the greatest number of DE-lncRNAs in the comparison of “21 d vs. 27 d”, including 389 up-regulated and 703 down-regulated DE-lncRNAs (Fig. 3C). Additionally, only 8 DE-lncRNAs were detected in all three comparisons, whereas 19,865 and 71 lncRNAs were uniquely differentially expressed in the comparison of “21 d vs. 24 d”, “21 d vs. 27 d” and “24 d vs. 27 d”, respectively (Fig. 3F).

### Functional enrichment of differentially expressed mRNAs, MiRNAs and LncRNAs

**Fig. 4:**
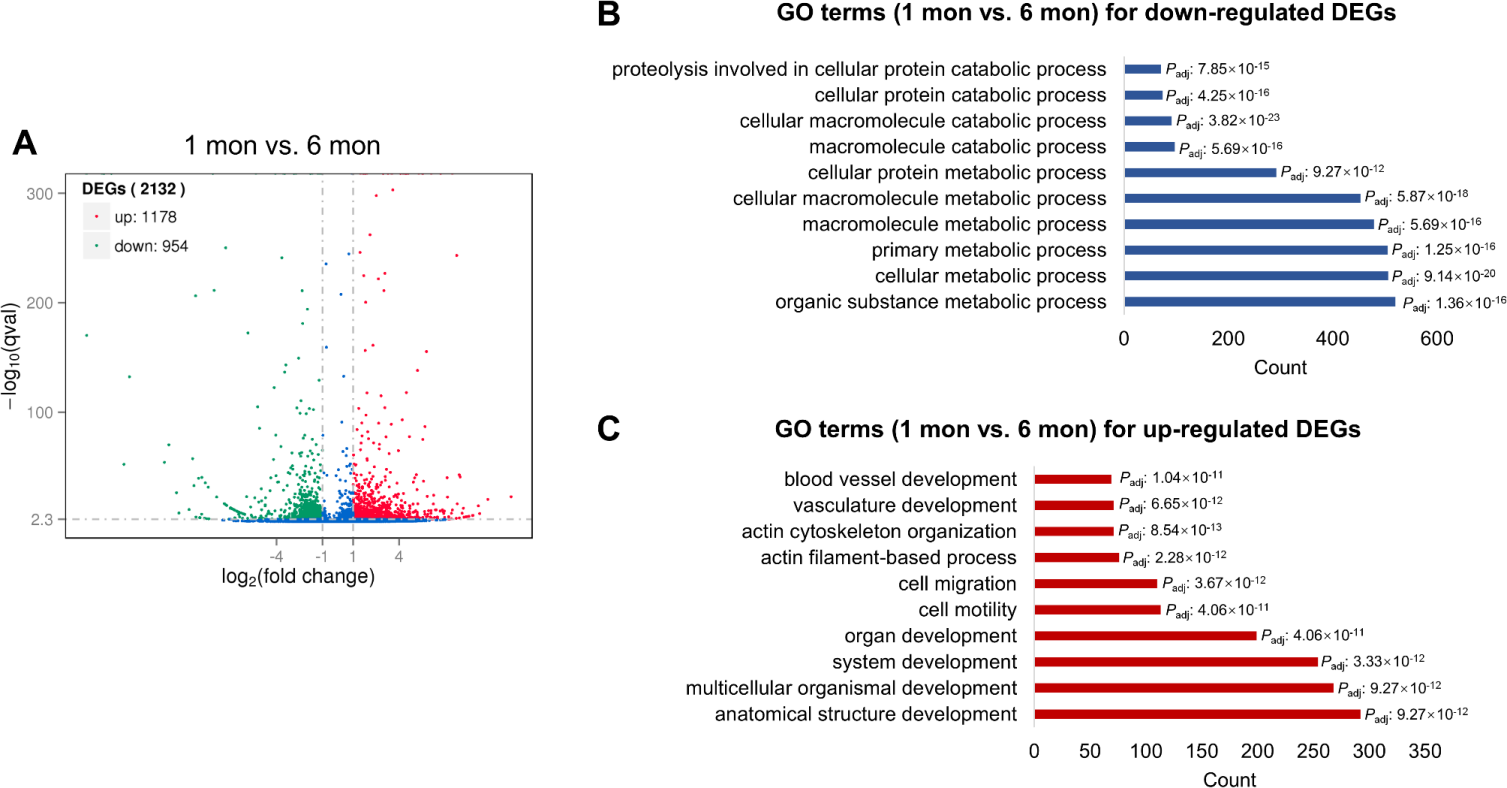
Differential expression analysis of mRNAs between 1 month and 6 months. **A** Volcano plots of up-regulated and down-regulated DEGs between 1 month and 6 months. The blue dots represent non-significantly DEGs. **B**, **C** The top 10 GO terms enriched from down- and up-regulated DEGs in the “1 mon vs. 6 mon” comparison. The blue bar represents terms of down-regulated genes (**B**) and the red bar represents terms of up-regulated genes (**C**)

To investigate the functions of the differentially expressed genes, we performed Gene Ontology (GO) and Kyoto Encyclopedia of Genes and Genomes (KEGG) analyses for comparisons of each sequencing dataset separately (Tables S5-S9). We mainly focused on the comparison of which harbored the greatest number of DE-RNAs. We found the highest number of DE genes in the “1 mon vs. 6 mon” comparison, which included 1,178 up-regulated and 954 down-regulated DE genes (Fig. 4A). We then performed functional enrichment for these genes and found that the functions of the up- and down-regulated DEGs were different (Fig. 4B and C). For example, up-regulated DE genes were associated with the GO terms “action filament-based process” (e.g., *CACNA2D1*, *AKAP9* and *RYR2*) and “anatomical structure development” (e.g., *HOMER1*, *MYOC* and *GSK3B*) (Fig. 4B, Table S5 and Table S6), whereas down-regulated DEGs were largely associated with macromolecule metabolic processes (e.g., *ATF3*, *JAK2* and *IDE*) (Fig. 4C).

**Fig. 5:**
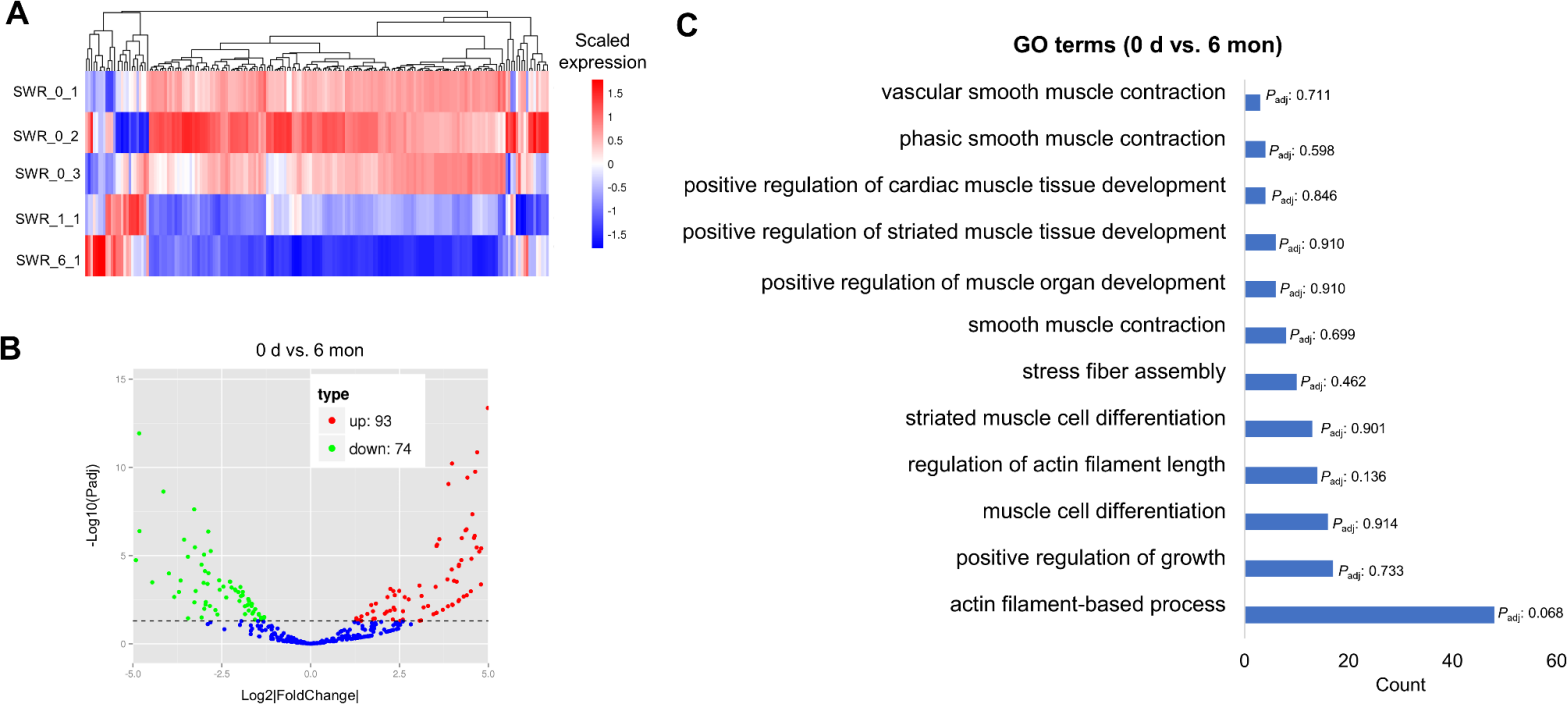
Differential expression analysis of miRNAs between 0 day and 6 months. **A** Heatmap showing the expression of DE-miRNAs in all the miRNA samples. **B** Volcano plots of up-regulated and down-regulated between 0 day and 6 months. The blue dots represent non-significant DEGs. **C** Muscle-related GO terms enriched from DE-miRNAs in the “0 d vs. 6 mon” comparison

**Fig. 6:**
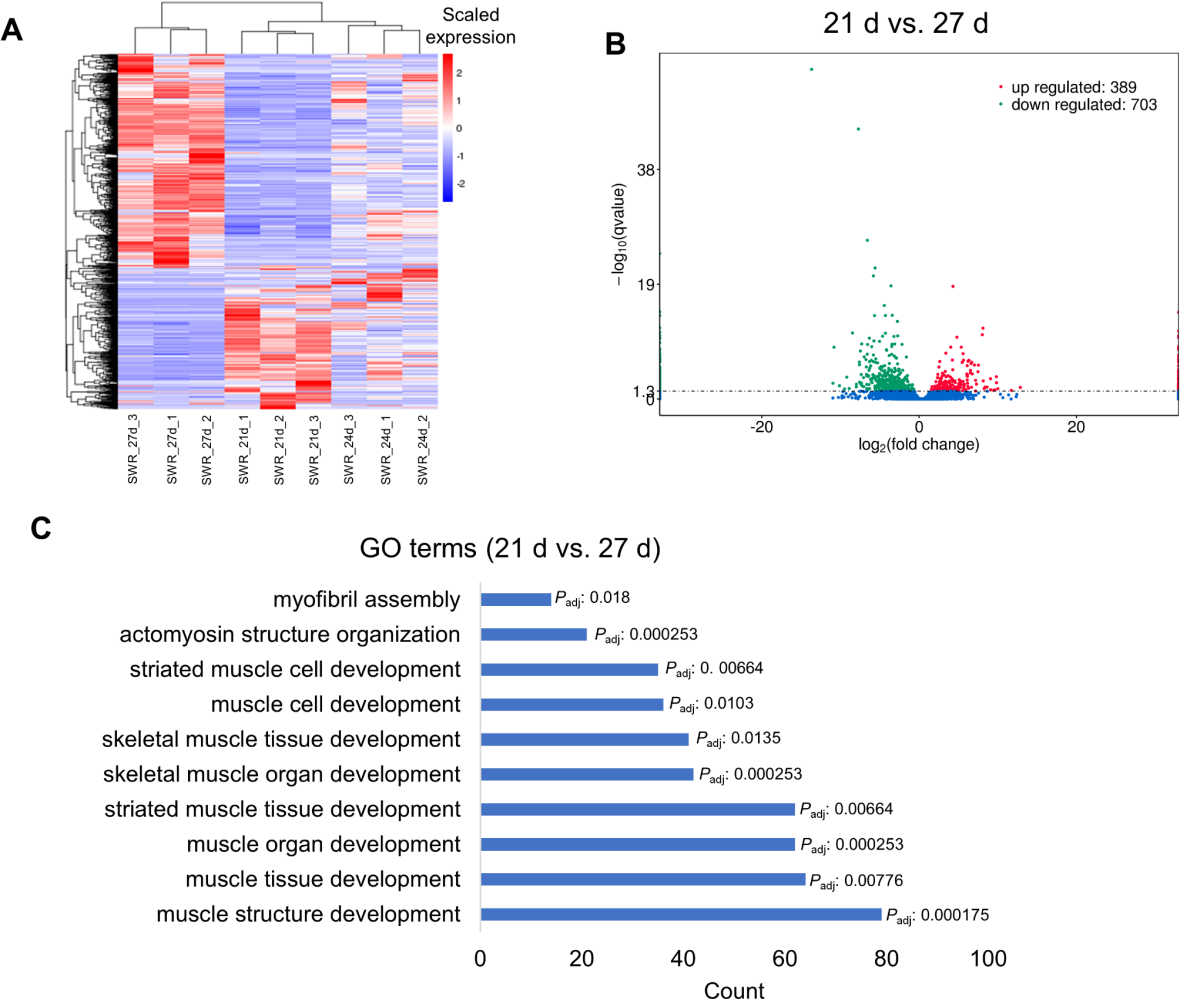
Differential expression analysis of lncRNAs between 21 days and 27 days. **A** Heatmap showing the expression of DE-lncRNAs in all the lncRNA samples. **B** Volcano plots of up-regulated and down-regulated between 21 days and 27 days. The blue dots represent non-significant DEGs. **C** The top 10 GO terms enriched DE-lncRNAs in the “21 d vs. 27 d” comparison

For miRNA-seq, the hierarchical clustering of the DE-miRNAs heatmap showed their expression dynamics at three time points (Fig. 5A). We detected the greatest number of DE-miRNAs in the comparison of “0 d vs. 6 mon” (Fig. 5B). The top 10 GO terms were associated with basic biological processes (Table S7), such as “transport” (e.g., *SPTBN2* and *TGFB3*), “single-organism transport” (e.g., *CAMK1* and *CLCN3*) and “establishment of localization” (e.g., *CHMP7* and *RTN2*). Besides, we also found several muscle-related GO terms (Fig. 5C), such as “positive regulation of growth” (e.g., *SMO*, *TBX2* and *WNT3 A*) and “striated muscle cell differentiation” (e.g., *MYOG*, *MYPN* and *EDN1*). For lncRNA-seq, we examined the expression of the DE-lncRNAs and observed large changes in the comparison of “21 d vs. 27 d” (Fig. 6A), which obtained the greatest number of DE-lncRNAs (Fig. 6B). Surprisingly, we found that the top 10 GO terms from this comparison were closely related to muscle development (Fig. 6C, Table S8, Table S9), such as “muscle structure development” (e.g., *TNNC1*, *MYF6* and *MYL3*), “muscle organ development” (e.g., *ACTN3*, *IGF1* and *MYLK2*) and “skeletal muscle organ development” (e.g., *MSTN*, *MYOG* and *CXCL9*).

### Interaction of differentially expressed LncRNAs between key time points

**Fig. 7:**
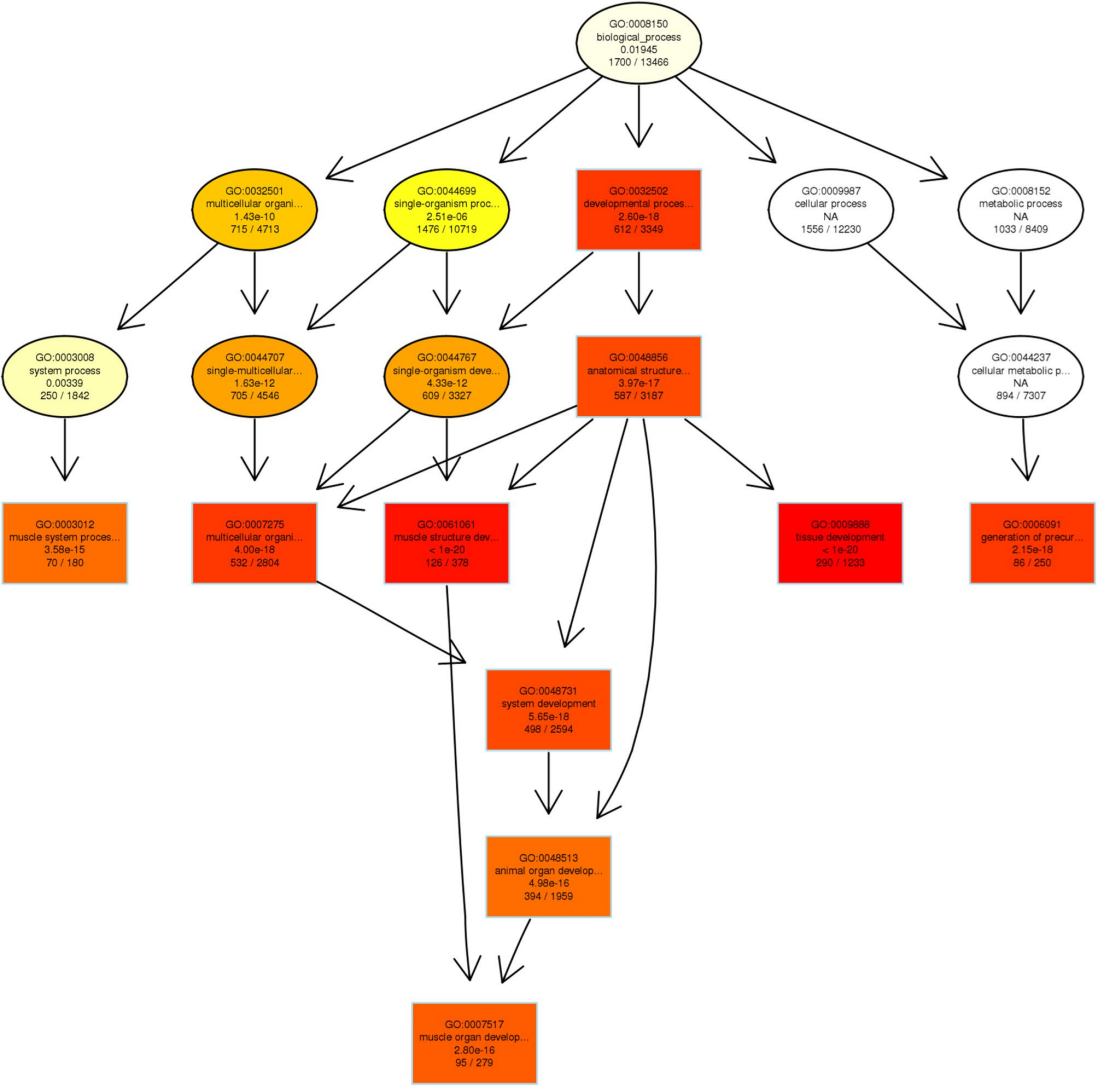
Directed acyclic graph (DAG) plot of the enriched GO terms for the target genes of the DE-lncRNAs

To address how miRNAs and lncRNAs interact with their target genes (mRNAs) to regulate rabbit muscle development, we predicted potential target genes and investigated their functions. The results of the above differential expression analysis, we found the greatest number of DE-lncRNAs in the comparison of “21 d vs. 27 d”, and their function was associated with muscle growth. Previous studies have confirmed that lncRNAs regulate the expression of neighboring protein-coding genes through *cis*-acting mechanisms [28]. Thus, we performed co-expressed gene analysis between lncRNAs and mRNAs with a correlation coefficient > 0.95 as the threshold and concentrated on this comparison. The directed acyclic graph (DAG) plot for GO terms enriched from the target genes of the DE-lncRNAs showed complex regulatory network (Fig. 7).

## Discussion

The growth and development of muscle is a dynamic and complex process that largely affects the meat production of livestock. In this study, we systemically investigated the complex regulatory landscape of muscle development by conducting a comprehensive sequencing analysis of mRNAs, miRNAs, and lncRNAs. Myogenesis is a highly coordinated developmental process. Our transcriptome data included three time points that can cover different stages. For mRNAs and miRNAs, we selected 0 day, 1 month and 6 months to represent the fetal, child, and adult periods of rabbits, respectively [29, 30]. The total number of fibers is fixed during the first postnatal month of myogenesis in rabbits [30]. In particular, longissimus dorsi is no longer expressed 27 days after birth [31]. Hence we selected 21 days, 24 days and 27 days to explore lncRNA changes. By performing PCA and HCA analyses, we found that samples in the same group tended to cluster together (Fig. 2) and verified the reliability of the data for downstream analysis.

Differential expression analysis revealed that gene expression changes corresponded to distinct developmental stages. The comparison of mRNA and miRNA datasets revealed a total of 2,995 DE genes and 305 DE-miRNAs, respectively, with notable variations in expression patterns. The lncRNA-seq analysis identified 1,211 DE-lncRNAs, and the most significant changes were observed between 21 d and 27 d. We found gene *PAX7* and *MEF2* were down-regulated in the comparison of “21 d vs. 27 d” (Table S9) only. *PAX7* is expressed in satellite cells during the postnatal development of rabbits and plays an important role in muscle regeneration and repair. Similar functions have also been reported in mice and chickens [32]. *MEF2* serves as a transcription factor that can regulate muscle fiber identity and maintenance. The down-regulation of these two genes suggested that the number of fibers stopped increasing in the first postnatal month in rabbits [30]. Moreover, muscle also can change their functional characteristics in response to the physiological stage (i.e., growing, maintaining and senescing) of animals. For instance, the dynamic expression of miRNAs and lncRNAs in 7 different periods in goats was reported to affect skeletal muscle development [33, 34]. In black Muscovy duck, differentially expressed genes like *MyoG*, *FBXO1*, *MEF2 A*, and *FoxN2* in leg muscle were enriched in growth-related biological processes [35]. These findings suggest that myogenesis is relatively conserved among species.

Functional enrichment analysis of the DE mRNAs and miRNAs shed light on their biological roles, with a particular emphasis on the “1 mon vs. 6 mon” comparison for mRNAs. This analysis revealed that the up-regulated DE genes were involved in processes such as actin filament-based movement (e.g., *CACNA2D1*, *AKAP9* and *RYR2*) and anatomical structure development (e.g., *HOMER1*, *MYOC* and *GSK3B*) (Fig. 4B), while down-regulated DE genes were predominantly associated with macromolecule metabolism (e.g., *ATF3*, *JAK2* and *IDE*) (Fig. 4C). miRNA-seq analysis revealed a dynamic expression pattern, with the “0 d vs. 6 mon” comparison yielding the greatest number of DE-miRNAs (Fig. 3B). The GO terms associated with these miRNAs pointed towards fundamental biological processes and muscle-specific functions (Fig. 5C), such as positive regulation of growth (e.g., *SMO*, *TBX2* and *WNT3 A*) and striated muscle cell differentiation (e.g., *MYOG*, *MYPN* and *EDN1*). Notably, we detected the greatest number of DE-lncRNAs in the comparison of “21 d vs. 27 d” (Fig. 3C), and the functions of these DE-lncRNAs were closely associated with muscle growth (Fig. 6C). MyoG and Myf5, these two important MRFs were largely enriched in multiple muscle-related GO terms, such as “muscle organ development” and “striated muscle tissue development” (Fig. 6, Table S9) in the comparison of “21 d vs. 27 d”. In addition, interaction analysis between DE-lncRNAs and their target genes unveiled a complex regulatory network. Our co-expression analysis used a stringent threshold of correlation > 0.95, and the subsequent DAG plot of GO terms enriched from the target genes clearly showed the intricate interplay between lncRNAs and mRNAs in modulating muscle development (Fig. 7).

Furthermore, the above mentioned target genes play important roles in muscle development. Myogenic cell specification and differentiation are determined by the master transcription factor MyoD in concert with other myogenic regulatory factors (MRFs) [36]. In particular, MyoG plays a central role in the terminal differentiation of myoblasts into mature muscle fibers [37]. Besides, MyoG is regulated by growth factor signaling pathways, especially the IGF1 pathway. IGF1 can enhance the expression of above-mentioned MRFs by activating the PI3 K/Akt pathway, which works collectively to promote muscle growth and repair [38]. Previous study reported that the interplay between IGF signaling and MRFs can also be modulated by miRNAs like miR- 1 and miR- 133 [39]. These results suggest dramatic lncRNA changes in the expression of myosin heavy chain isoforms in the first postnatal month. Besides, gene *MYOC* encodes a protein that belongs to the olfactomedin family and is expressed in various tissues, including skeletal muscle. Although its specific function in muscle is not well characterized, it is known to be involved in the development and maintenance of muscle mass, potentially through interactions with other muscle regulatory factors [40]. *WNT3 A* belongs to the Wnt signaling pathway, this pathway regulates muscle formation and the maintenance of adult tissue homeostasis [41, 42]. Besides, *JAK2* is part of the JAK-STAT signaling pathway. This pathway is involved in the regulation of muscle growth and differentiation, with *JAK2* being a key mediator of these processes. Mutations in *JAK2* have been associated with myeloproliferative disorders, which can have secondary effects on muscle function [43]. The *RYR2* gene encodes the type 2 ryanodine receptor, a calcium release channel found in the sarcoplasmic reticulum of muscle cells. It is essential for the regulation of calcium ions during muscle contraction and relaxation. Dysfunction of *RYR2* has been implicated in various muscle diseases, including malignant hyperthermia and central core disease, highlighting its importance in muscle function [44]. Overall, these genes and their protein products are integral to the complex processes of muscle growth, development, and maintenance.

The present study has several limitations. First, the small sample size for each time point has potential impact on expression patterns, although unrelated factors were controlled. Previous studies have reported that sufficient biological replicates enable to capture of authentic biological variability and avoid technical artifacts [45, 46]. Proper statistical models or decreased multiple comparisons can also reduce false positive errors. In addition, quantitative polymerase chain reaction (qPCR) is one of the most reliable methods for validating gene expression [47] and has been widely used in transcriptome research. Due to the restriction of sample collection, our study only explored expression patterns during muscle development based on sequencing data. In future work, we will cautiously consider the impact of sample size and add a qPCR experiment to validate the expression of candidate genes. Second, tissues were collected from males only. Thus, sex-based differences in muscle expression cannot be addressed. Future research could benefit from the increased sample size of both male and female rabbits and the use of different layers of multi-omics data (e.g., chromatin accessibility and DNA methylation). It will greatly enhance the understanding of the regulation of rabbit muscle development.

## Conclusions

This study provides a comprehensive overview of the transcriptomic changes that occur during muscle development. The identification and functional annotation of DE genes, miRNAs, and lncRNAs provide valuable insights into the molecular mechanisms underlying this process. These findings pave the way for investigations into the role of non-coding RNAs in muscle biology.

## Supplementary Information

Supplementary Material 1.

## Data Availability

The datasets generated in this study are available in the Sequence Read Archive (SRA) with the primary accession code PRJNA1173484 of the NCBI database.

## Abbreviations

miRNAs: Micro RNA
lncRNAs: Long non-coding RNAs
SWR: Sichuan white rabbit
PBS: Phosphate buffered saline
TPM: Transcripts per million
DE: Differential expressed
GO: Gene Ontology
CPC: Coding potential calculator
CNCI: Coding-non-coding index
Pfam: Protein families database
KEGG: Kyoto Encyclopedia of Genes and Genomes
DAG: Directed acyclic graph
qPCR: quantitative polymerase chain reaction
